# The antitumour activity of 5,6-dimethylxanthenone-4-acetic acid (DMXAA) in TNF receptor-1 knockout mice

**DOI:** 10.1038/sj.bjc.6600479

**Published:** 2002-08-12

**Authors:** L Zhao, L-M Ching, P Kestell, B C Baguley

**Affiliations:** Auckland Cancer Society Research Centre, Faculty of Medical and Health Sciences, The University of Auckland, Private Bag 92019, Auckland, New Zealand

**Keywords:** antivascular, tumour necrosis factor, serotonin

## Abstract

5,6-dimethylxanthenone-4-acetic acid, a novel antivascular anticancer drug, has completed Phase I clinical trial. Its actions in mice include tumour necrosis factor induction, serotonin release, tumour blood flow inhibition, and the induction of tumour haemorrhagic necrosis and regression. We have used mice with a targeted disruption of the tumour necrosis factor receptor-1 gene as recipients for the colon 38 carcinoma to determine the role of tumour necrosis factor signalling in the action of 5,6-dimethylxanthenone-4-acetic acid. The pharmacokinetics of 5,6-dimethylxanthenone-4-acetic acid, as well as the degree of induced plasma and tissue tumour necrosis factor, were similar in tumour necrosis factor receptor-1^−/−^ and wild-type mice. However, the maximum tolerated dose of 5,6-dimethylxanthenone-4-acetic acid was considerably higher in tumour necrosis factor receptor-1^−/−^ mice (>100 mg kg^−1^) than in wild-type mice (27.5 mg kg^−1^). The antitumour activity of 5,6-dimethylxanthenone-4-acetic acid (25 mg kg^−1^) was strongly attenuated in tumour necrosis factor receptor-1^−/−^ mice. However, the reduced toxicity in tumour necrosis factor receptor-1^−/−^ mice allowed the demonstration that at a higher dose (50 mg kg^−1^), 5,6-dimethylxanthenone-4-acetic acid was curative and comparable in effect to that of a lower dose (25 mg kg^−1^) in wild-type mice. The 5,6-dimethylxanthenone-4-acetic acid -induced rise in plasma 5-hydroxyindoleacetic acid, used to reflect serotonin production in a vascular response, was larger in colon 38 tumour bearing than in non-tumour bearing tumour necrosis factor receptor-1^−/−^ mice, but in each case the response was smaller than the corresponding response in wild-type mice. The results suggest an important role for tumour necrosis factor in mediating both the host toxicity and antitumour activity of 5,6-dimethylxanthenone-4-acetic acid, but also suggest that tumour necrosis factor can be replaced by other vasoactive factors in its antitumour action, an observation of relevance to current clinical studies.

*British Journal of Cancer* (2002) **87**, 465–470. doi:10.1038/sj.bjc.6600479 www.bjcancer.com

© 2002 Cancer Research UK

## 

The tumour vasculature is a promising new target in cancer therapy, and a number of antitumour agents have been identified that either inhibit tumour angiogenesis (Baillie *et al*, 1995) or destroy the tumour's existing blood supply (Baguley, 2001). Tumour necrosis factor (TNF), a pleiotropic cytokine that is produced mainly by activated monocytes/macrophages, neutrophils, and T cells (Balkwill, 1989), is thought to exert its antitumour effect mainly through an antivascular mechanism. Flavone-8-acetic acid (FAA), and its more potent analogue 5,6-dimethylxanthenone-4-acetic acid (DMXAA), developed in this laboratory, are low molecular weight antivascular agents that appear to exert their antitumour effects at least partly through the induction of TNF (Mace *et al*, 1990; Philpott *et al*, 1995). FAA proved to be inactive in clinical studies (Kerr and Kaye, 1989), but DMXAA has shown evidence of activity in a Phase I clinical trial (Jameson *et al*, 2000).

In mice DMXAA induces, in addition to TNF, serotonin (Baguley *et al*, 1997), nitric oxide (Thomsen *et al*, 1991), interferons, interferon regulatory factors, and IP-10 (Cao *et al*, 2001). Like FAA, DMXAA causes protracted inhibition of blood flow in murine tumours (Zwi *et al*, 1994; Lash *et al*, 1998) and induces extensive tumour haemorrhagic necrosis that is similar to that induced by TNF (Rewcastle *et al*, 1991). *In situ* hybridisation studies indicate that both host and tumour cells within murine colon 38 tumours express TNF mRNA after DMXAA treatment (Joseph *et al*, 1999). An administration schedule of DMXAA (two doses, 3 days apart) that increases TNF production also improves antitumour activity (Philpott *et al*, 1995). Co-administration of thalidomide with DMXAA increases intratumoural TNF synthesis and concomitantly increases cure rate (Ching *et al*, 1995; Browne *et al*, 1998; Cao *et al*, 1999).

While the above reports indicate that TNF induction is important for the antitumour action of DMXAA, there is quite extensive evidence that TNF-independent mechanisms may contribute to its antitumour effect. A significant decrease in tumour blood flow is observed within 1 h of administration of DMXAA, before detectable induction of cytokines (Zwi *et al*, 1994; Lash *et al*, 1998). Induced endothelial cell apoptosis in tumour tissue occurs as early as 15 min after DMXAA administration (Ching *et al*, 2002). Co-administration of anti-TNF antibodies only partially reverses the blood flow and antitumour effects of DMXAA (Browne *et al*, 1998). Finally, DMXAA induces tumour haemorrhagic necrosis in TNF knockout hosts (Ching *et al*, 1999).

The activities of TNF are mediated by binding to two receptors: TNFR1 (p55), and TNFR2 (p75) (Aggarwal and Natarajan, 1996). TNFR1 is ubiquitous except in erythrocytes and unstimulated T cells, while TNFR2 is often more abundant on cells of haemopoietic lineage and is also expressed on endothelium (Slowik *et al*, 1993). Recent studies suggest that TNFR1-expressing endothelial cells of the tumour vasculature are the targets of TNF-induced necrosis (Stoelcker *et al*, 2000). In the present study, we have sought to evaluate the role of TNF in the host response to DMXAA by utilising mice with targeted disruption of TNF receptor I gene (TNFR1^−/−^) as recipients for the (TNF positive) colon 38 carcinoma. We have examined four end-points, toxicity, tumour growth delay, induction of tumour haemorrhagic necrosis, and serotonin production. Because DMXAA pharmacokinetics are of major importance in the interpretation of experiments combining DMXAA with drugs such as thalidomide (Kestell *et al*, 2000) and cyproheptadine (Zhao *et al*, 2001), we also measured drug pharmacokinetics in wild-type (WT) and TNFR1^−/−^ mice.

## MATERIALS AND METHODS

### Mice and tumour model

Breeding stocks of C57Bl/6 and TNFR1^−/−^ mice on a C57Bl background were obtained from Jackson Laboratories, Bar Harbor, Maine, USA. WT and TNFR1^−/−^ mice were bred in the Animal Resources Unit, University of Auckland (TNFR1^−/−^ mice under specific pathogen-free conditions). All experiments were approved by the University of Auckland Animal Ethics Committee, and conformed to the Guidelines for the Welfare of Animals in Experimental Neoplasia, as set out by the United Kingdom Co-ordinating Committee on Cancer Research. Mice were used between 6 and 12 weeks of age and the colon 38 tumour was implanted subcutaneously in mice that had been anaesthetised by intraperitoneal (i.p.) administration of pentobarbitone (87 mg kg^−1^). DMXAA was synthesised in this laboratory (Rewcastle *et al*, 1991), dissolved in sterile saline, protected from light (Rewcastle *et al*, 1990), and administered i.p. (10 μl g^−1^ body weight).

### Tumour growth delay and tumour necrosis determination

Mice were treated i.p. with DMXAA when the tumours were approximately 5 mm in diameter. Tumour size was measured thrice weekly using callipers and the volumes calculated as 0.52×*a*^2^×*b*, where *a* and *b* were the minor and major tumour axes. The arithmetic means and standard error of the means were calculated for each time point, counting cured animals as having zero tumour volume, and expressed as fractions of the pre-treatment tumour volume. Growth delay was determined as the difference in the number of days required for the untreated and treated tumours to reach four times the pre-treatment volume. Tumour necrosis was measured in WT and TNFR1^−/−^ mice in which colon 38 tumours had been implanted and allowed to grow to a diameter of approximately 6 mm. Tumours were removed 24 h after drug treatment, fixed in 10% formalin, sectioned and stained with haematoxylin/eosin. Tumour necrosis was quantified microscopically using a grid system (Baguley *et al*, 1989).

### TNF assay

Mice were anaesthetised with halothane and bled from the ocular sinus. The blood was allowed to coagulate overnight on ice. After centrifugation of samples (2000 **g**, 30 min, 4°C) the serum was then removed and stored at −70°C. Tumour, spleen and liver tissues were homogenised in 1.5 ml of α-MEM medium using a tissue homogeniser. The homogenates were centrifuged (2000 **g**, 30 min, 4°C) and the supernatant was removed and re-centrifuged (14 000 **g**, 30 min at 4°C). Serum and supernatants from tissue homogenates were kept at −70°C until use. TNF was assayed using a commercially available ELISA kit (OptEIA Mouse TNF kit, PharMingen, San Diego, CA, USA) according to the manufacturer's directions.

### 5-hydroxyindoleacetic acid (5HIAA) assay

Blood samples (700–800 μl) from DMXAA-treated and untreated mice were collected from the ocular sinus into heparinised tubes during halothane anaesthesia, centrifuged, and the plasma was removed and mixed with 0.1 M HCl containing 0.01% ascorbic acid. 5HIAA concentrations were measured using automated solid phase extraction and high performance liquid chromatography (HPLC) as previously described (Zhao *et al*, 2001).

### DMXAA assay

Tumour-bearing mice (three per group) were treated with DMXAA (25 mg kg^−1^ i.p.). After 0.25, 1, 2, 3, 8 and 24 h, blood samples (700–800 μl) were collected from the ocular sinus into heparinised tubes during halothane anaesthesia, centrifuged, and the plasma was removed and stored at −20°C until analysis. DMXAA plasma concentrations were measured using automated solid phase extraction and high performance liquid chromatography (HPLC) as previously described (Kestell *et al*, 2000).

## RESULTS

### TNF production in response to DMXAA

The maximum tolerated doses (MTD) of DMXAA in TNFR1^−/−^ mice was determined and found to be much higher in TNFR1^−/−^ mice than in WT mice (Table 1

**Table 1:**
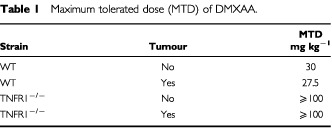
Maximum tolerated dose (MTD) of DMXAA

). The capacity of DMXAA to induce TNF was compared 2 h after treatment with DMXAA at doses of up to 100 mg kg^−1^. The maximal dose was considerably greater than the MTD in WT mice (Table 1), but could be used because it has been shown previously (Philpott *et al*, 1995) that no signs of distress occur within the time period of the experiment. DMXAA induced similar TNF activity in WT and TNFR1^−/−^ mice, with maximal activity at 75 mg kg^−1^ (Figure 1

**Figure 1:**
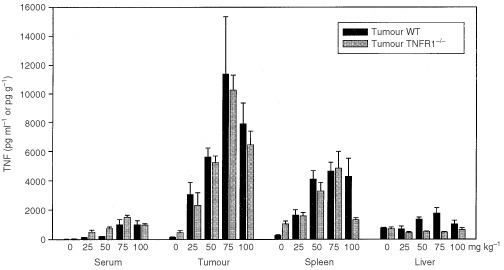
Dose response for TNF induction by DMXAA. TNF activity in serum, colon 38 tumour, spleen, and liver of WT and TNFR1^−/−^ mice, untreated or 2 h after treatment with a range of DMXAA doses, was measured by ELISA assay and expressed as the mean±s.e. (*n*=3).

). The TNF response in tumour tissue was considerably higher than that in serum. The response in liver tissue was small, while that in spleen was intermediate between those in tumour and liver (Figure 1).

### Antitumour activity

**Figure 2:**
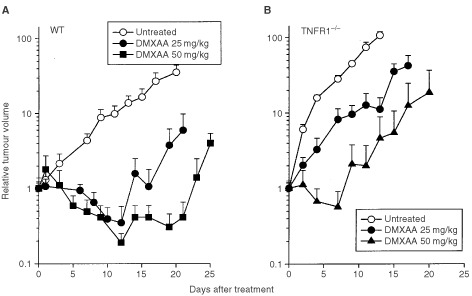
Growth of colon 38 tumours in WT (**A**) and TNFR1^−/−^ (**B**) mice. Tumor volumes in mice without treatment, or following treatment with DMXAA at doses of 25, 27.5 and 50 mg kg^−1^ were expressed as the mean±s.e. (*n*=5).

The responses to DMXAA of colon 38 tumours growing in WT and TNFR1^−/−^ mice were compared. In WT mice, DMXAA (25 and 27.5 mg kg^−1^) produced growth delays of 13 and 19 days, respectively, and cure rates of 40% (Figure 2A

; Table 2

**Table 2:**
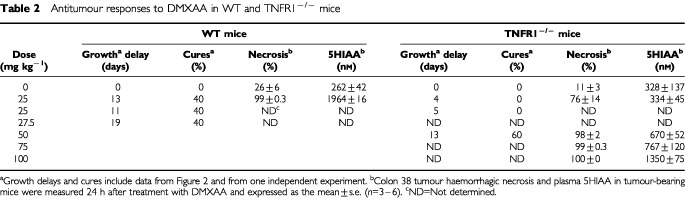
Antitumour responses to DMXAA in WT and TNFR1^−/−^ mice

). In TNFR1^−/−^ mice, DMXAA (25 mg kg^−1^) induced only a short tumour growth delay (4 days) with no cures. However, a higher dose (50 mg kg^−1^) induced a tumour growth delay of 13 days and a cure rate of 60% (Figure 2B; Table 2), similar to that induced by a dose of 25 mg kg^−1^ in WT mice.

### Induction of haemorrhagic necrosis

Colon 38 tumours growing in WT and TNFR1^−/−^ mice were removed and examined 24 h after treatment with DMXAA (25 mg kg^−1^). Tumours from untreated WT and TNFR1^−/−^ mice showed either little necrosis or central necrosis occupying up to 30% of the section area. Tumours from DMXAA-treated WT and TNFR1^−/−^ mice showed extensive areas of haemorrhagic necrosis that were identical in histological appearance. The proportion of tumour haemorrhagic necrosis in mice treated with DMXAA (25 mg kg^−1^) was 99% in WT and 76% in TNFR1^−/−^ mice, but at higher doses, tumours in TNFR1^−/−^ mice exhibited a similar degree of necrosis (Table 2).

### 5HIAA response

**Figure 3:**
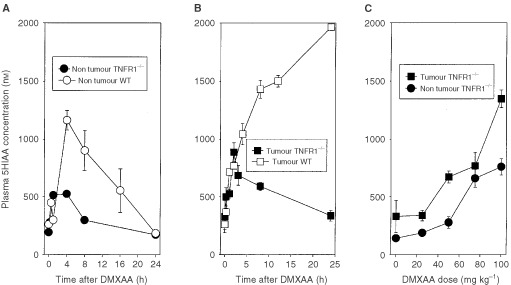
Plasma 5HIAA responses following administration of DMXAA. (**A**) Time course of 5HIAA response of non-tumour bearing WT mice treated with DMXAA (30 mg kg^−1^) and non-tumour bearing TNFR1^−/−^ mice treated with DMXAA (25 mg kg^−1^). (**B**) Time course of 5HIAA response of colon 38-tumour bearing TNFR1^−/−^ mice treated with DMXAA (25 mg kg^−1^). Previously published data (Zhao *et al*, 2002) for tumour bearing WT mice treated with DMXAA (25 mg kg^−1^) is shown for comparison. (**C**) Dose dependence of 5HIAA response (24 h after administration of DMXAA) for TNFR1^−/−^ mice without or with colon 38 tumours. Each point represents the mean±s.e. (*n*⩾3).

Previous studies in our laboratory have shown that treatment of mice with antivascular agents induces the accumulation in plasma of serotonin and its metabolite 5HIAA (Baguley *et al*, 1997). 5HIAA is the preferred marker for routine assay because of its greater stability (Kestell *et al*, 2001). Administration of DMXAA (25 mg kg^−1^) to non-tumour bearing WT mice produced a significant (*P*<0.001) increase in 5HIAA after 30 min, followed by a further increase to a maximum after 4 h and a decrease thereafter. Administration to non-tumour TNFR1^−/−^ mice also produced a significant increase in 5HIAA after 15 min (*P*=0.001) and 60 min (*P*<0.001) but no further increase thereafter (Figure 3A

). Administration of DMXAA to colon 38 tumour bearing WT mice caused plasma 5HIAA concentrations to rise continuously over the 24-h period of measurement, as shown previously (Zhao *et al*, 2002). In contrast, administration of DMXAA to tumour bearing TNFR1^−/−^ mice caused plasma 5HIAA to increase over 3 h but to decline at later times (Figure 3B). The dose response of plasma 5HIAA in TNFR1^−/−^ mice was measured 24 h after administration of DMXAA, and was found to increase with dose up to 100 mg kg^−1^. The response was generally greater in tumour bearing mice than in non-tumour bearing mice (Figure 3C).

### Pharmacokinetics of DMXAA

**Figure 4:**
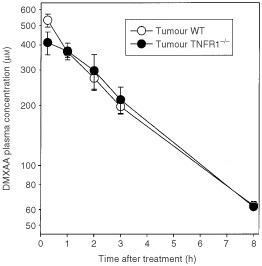
Plasma DMXAA concentration-time profiles in WT and TNFR1^−/−^ colon 38-bearing mice. Concentrations were measured up to 8 h after administration of DMXAA (25 mg kg^−1^) and expressed as the mean±s.e. (*n*=3).

To determine whether the difference in antitumour activity of DMXAA in WT and TNFR1^−/−^ mice was influenced by altered DMXAA pharmacokinetics, we compared DMXAA plasma concentration-time profiles in WT and TNFR1^−/−^ colon 38 tumour-bearing mice up to 8 h after DMXAA administration (25 mg kg^−1^) (Figure 4

). The plasma Cmax values in WT and TNFR1^−/−^ mice, 530±39 and 411±53 μmol l^−1^ respectively, as well as plasma concentrations at other times, were not significantly different. The corresponding plasma half-lives were 2.6 h and 2.8 h respectively, and the areas under the concentration-time curves (AUC) were 1623 and 1609 μmol.h l^−1^, respectively.

## DISCUSSION

One of the most unusual properties of DMXAA is its ability, as a low molecular weight drug, to induce the cytokine TNF in both plasma and tumour tissue (Philpott *et al*, 1995; Cao *et al*, 1999). The results described here provide two novel findings on the role of TNF in this action. The first is that the MTD of DMXAA is much higher in TNFR1^−/−^ mice (Table 1), suggesting a critical role for TNF in host toxicity. The second is that DMXAA can induce TNFR1-independent cures of colon 38 tumours. The results extend two previous studies, one using anti-TNF antibody (Browne *et al*, 1998) and one using TNF knockout mice (Ching *et al*, 1999), which raised the question of whether TNF is essential for antitumour activity.

The mechanism of DMXAA host toxicity has not yet been clearly defined but studies of tissue pathology in mice treated with DMXAA indicate that vascular damage can occur, particularly in tissues with a low vascular density such as peripheral lymphoid tissues, the thymus gland and the uterus (Zwi *et al*, 1990). Mice administered DMXAA at the MTD may exhibit sluggish movement, reduced body temperature and an increased haematocrit (unpublished data), and the lack of such symptoms in TNFR1^−/−^ mice suggests that TNFR1 receptors on the vascular endothelium, may be a major mediator of this toxicity of DMXAA. The toxic effects of DMXAA in WT mice are consistent with changes to normal vascular endothelium causing reduced peripheral blood flow and oedema. Possible mechanisms include TNF-induced increases in vascular permeability (Royall *et al*, 1989) and TNF-induced apoptosis of normal endothelial cells, which has been observed both *in vitro* and *in vivo* (Polunovsky *et al*, 1994; Messmer *et al*, 1999).

The induction of host antivascular effects might arise from a direct effect of DMXAA on the vascular endothelium, for instance by changed vascular permeability (Baguley, 2001) or by induced apoptosis of vascular endothelial cells (Ching *et al*, 2002). In addition, the antivascular effects may occur indirectly through the production of TNF (Baguley, 2001). One feature of the action of a variety of antivascular drugs is their induction of serotonin release into plasma (Baguley *et al*, 1997). The use of TNFR1^−/−^ mice, together with monitoring of plasma 5HIAA as a more stable metabolite of serotonin (Kestell *et al*, 2001), provides an opportunity to estimate the relative contributions of the direct and indirect effects of DMXAA. The similarity of DMXAA pharmacokinetics in WT and TNFR1^−/−^ mice (Figure 4), together with the similarity of intratumoural TNF production at the same DMXAA dose (Figure 1), are important for this comparison to be made. As shown in Figure 2A, DMXAA induces a plasma 5HIAA response in TNFR1^−/−^ mice at early times (within 15 min), indicating a TNF-independent vascular effect and consistent with the hypothesis that DMXAA can cause direct damage to the vascular endothelium (Ching *et al*, 2002). The 5HIAA response in WT mice also occurs early, but this is followed by a larger maximal response, consistent with it being a composite of both direct and TNF-mediated effects.

The 5HIAA responses of tumour-bearing TNFR1^−/−^ mice also provide information on the role of vascular effects in the antitumour response to DMXAA. As shown in Figure 2B, the response of WT mice to DMXAA (25 mg kg^−1^) is sustained for at least 24 h, consistent with the presence of an extended, tumour-specific vascular response. This sustained response is lacking in TNFR1^−/−^ mice treated at the same dose, in agreement with the reduced induction of tumour haemorrhagic necrosis and smaller induced tumour growth delay (Figure 2; Table 2). The results support the concept that the late, tumour-specific vascular response in WT mice treated at this dose is TNF-dependent. TNF may increase vascular permeability (Royall *et al*, 1989) as well as induce endothelial cell apoptosis (Polunovsky *et al*, 1994; Messmer *et al*, 1999), possibly mediated by reduced αvβ3 integrin-mediated tumour endothelial cell adhesion (Ruegg *et al*, 1998). A higher dose of DMXAA does induce tumour regressions in TNFR1^−/−^ mice (Table 2) and although time courses for 5HIAA production at higher doses have not been carried out, the dose response measured after 24 h increases markedly (Figure 2C), consistent with the generation of a sustained 5HIAA response in TNFR1^−/−^ mice at high dose.

In conclusion, the demonstration that at a dose of 50 mg kg^−1^, DMXAA demonstrates excellent antitumour activity in TNFR1^−/−^ mice without host toxicity (Figure 2; Table 1) suggests that other cytokines or vasoactive agents are induced by DMXAA and can substitute for TNF. The nature of the agent(s) involved is currently unknown, but other agents known to induce tumour haemorrhagic necrosis include interferons α/β (Dvorak and Gresser, 1989), interleukin-1α (Johnson *et al*, 1991), IP-10 (Sgadari *et al*, 1996), serotonin (Manda *et al*, 1988) and nitric oxide (Fukumura *et al*, 1997). DMXAA is known to induce interferons (Pang *et al*, 1998), IP-10 (Cao *et al*, 2001), serotonin (Baguley *et al*, 1993, 1997) and nitric oxide (Thomsen *et al*, 1991). Phase I trials of DMXAA show evidence of decreased tumour blood flow (Rustin *et al*, 1998; Jameson *et al*, 2000) and increased plasma 5HIAA (Kestell *et al*, 2001), but only a small increase in plasma nitrate and no increase in plasma TNF (Jameson *et al*, 2000). It is possible that other DMXAA-inducible cytokines are involved in humans, and their identification is an important consideration in future clinical trials.
